# Dopamine D2 Receptor Signaling Attenuates Acinar Cell Necroptosis in Acute Pancreatitis through the Cathepsin B/TFAM/ROS Pathway

**DOI:** 10.1155/2022/4499219

**Published:** 2022-07-26

**Authors:** Zengkai Wu, Xiao Han, Jingpiao Bao, Bin Li, Jie Shen, Pengli Song, Qi Peng, Xingpeng Wang, Guoyong Hu

**Affiliations:** ^1^Department of Gastroenterology, Shanghai General Hospital, Shanghai Jiao Tong University School of Medicine, Shanghai, China; ^2^Shanghai Key Laboratory of Pancreatic Disease, Institute of Pancreatic Disease, Shanghai Jiao Tong University School of Medicine, Shanghai, China

## Abstract

Acute pancreatitis (AP) is an inflammatory disease that is associated with trypsinogen activation, mitochondrial dysfunction, cell death, and inflammation. Dopamine D2 receptor (DRD2) plays an essential role in alleviating AP, while it is unclear whether it is involved in regulating acinar cell necroptosis. Here, we found that DRD2 agonist quinpirole alleviated acinar cell necroptosis via inhibiting cathepsin B (CTSB). Moreover, CTSB inhibition by CA-074Me ameliorated AP severity by reducing necroptosis. Notably, knockdown of TFAM reversed the therapeutic effect of either quinpirole or CA-074Me. We identified a new mechanism that DRD2 signaling inhibited CTSB and promoted the expression of mitochondrial transcription factor A(TFAM), leading to reduction of ROS production in AP, which attenuated acinar cell necroptosis ultimately. Collectively, our findings provide new evidence that DRD2 agonist could be a new potential therapeutic strategy for AP treatment.

## 1. Introduction

Acute pancreatitis (AP) is one of most common and potentially life-threatening gastrointestinal disorders, with an increasing global incidence [1–5]. Over the past decade, mortality rate of AP has declined [4]. However, the mortality in severe acute pancreatitis (SAP) characterized by systemic inflammatory response syndrome (SIRS) and persistent multi-organ failure (MOF) is still up to 30% [2]. Although great progress has been made in basic and clinical research of AP, there are still no effective treatment strategies to prevent AP. Pathological process of AP is closely associated with mitochondrial dysfunction, oxidative stress, premature protease activation, and cell death [3, 6–8]. Pancreatic acinar cell (PAC) death is a key pathological feature of AP, mainly manifested in two well-established forms: necrosis and apoptosis. Apoptosis is a favorable cell death mechanism, and induction of apoptosis rather than necrosis of pancreatic acinar cells can reduce the severity of AP [9, 10].

Accumulating evidence has shown that programmed process of necrosis, known as necroptosis, is triggered by death cytokines like tumor necrosis factor-*α* (TNF-*α*). Receptor-interacting protein 1, 3 (RIP1,3) interaction mediated necroptosis by forming a complex called necrosome, which activates downstream mixed-line kinase domain-like (MLKL) upon RIP3-dependent phosphorylation and ultimately leads to necroptotic cell death [11–15]. In addition, reactive oxygen species (ROS) mediates the positive feedback regulation in the programmed necrosis pathway [16]. Necroptosis has been implicated to be closely related to the pathogenesis of various inflammatory diseases, including atherosclerosis [17], acute kidney injury [18], inflammatory bowel diseases [19], and AP [20]. RIP3/MLKL signaling pathway plays a key role in acinar cell necroptosis and is associated with the severity and progression of AP. RIP3 depletion or MLKL deficiency markedly ameliorated caerulein-induced AP [11, 13].

Dopamine (DA), a catecholamine neurotransmitter, is found not only in the central nervous system but also in peripheral system, severing as a variety of distinct functions [21]. DA regulates its effects by activating dopamine receptors (DRDs), which are divided into two major families: D1-like family (D1 and D5 receptors) and D2-like family (D2, D3, D4 receptors) [22]. Recent researches have shown that DA plays an anti-inflammatory role via D1 and/or D2 receptor signaling [23–25]. Our previous studies showed that DA attenuated inflammation in two disparate models of AP specifically via D2 signaling [26, 27]. Furthermore, we also found that DRD2 activator quinpirole inhibited the processing and the activity of cathepsin B (CTSB) during two different models of AP [28]. Notably, Zhang et al. demonstrated that granulysin induced CTSB release from HeLa cells to decrease mitochondrial function, leading to necroptosis [29]. Recent studies suggest that although small amounts of cytosolic CTSB can trigger apoptotic pathway, excessive CTSB released from lysosomes into the cytosol can convert the cell death pathway to necrosis [30]. Taken together, we hypothesized that DRD2 modulates necroptosis of PACs, possibly through inhibition of CTSB release. However, the effect of DRD2 activation on PAC necroptosis and its mechanisms remains elusive.

Therefore, we firstly verified that DRD2 agonist quinpirole could significantly reduce necroptosis of PACs in vitro and in two experimental models of AP. In addition, quinpirole played a protective role on cell necroptosis at least partly through inhibiting CTSB. Moreover, inhibition of CTSB with CA-074Me attenuated pancreatic edema, inflammatory infiltration, and necrosis during two models of AP. Using isolated pancreatic acinar cells, we showed that inhibition of CTSB with CA-074Me decreased the expression of RIP3 and p-MLKL. Finally, knockdown of mitochondrial transcription factor A (TFAM) as a potential substrate of CTSB leaked in the cytosol could partly reverse the effect of CTSB inhibitor CA-074Me or DRD2 activator quinpirole. These findings revealed that DRD2 plays a crucial role in mediating necroptosis of PACs during AP.

## 2. Methods

### 2.1. Reagents and Antibodies

Caerulein (Cae; #HY-A0190) was purchased from MedChemExpress (Monmouth Junction, NJ, USA). Lipopolysaccharide (LPS, #L2880), cholecystokinin (CCK; #C2175), quinpirole (#Q102), Leu-Leu methyl ester hydrobromide (LLOMe,#L7393), and sodium taurocholate (NaT, #S0900000) were purchased from Sigma (St. Louis, MO, USA). Antibody against MLKL (#MABC604) was from Millipore (Billerica, MA, USA). Antibody against RIP3 (#sc-374639) was from Santa Cruz Biotechnology (Dallas, TX, USA). Antibody against p-MLKL (#ab196436) and RIP1 (#ab202985) were from Abcam (Cambridge, MA, USA). CA-074Methyl ester (CA-074Me, # S7420) was purchased from Selleck Chemicals (Houston, TX, USA).

### 2.2. Animal Models

Six~eight-week-old male C57BL6/J mice were obtained from Shanghai SLAC Laboratory Animal Co Ltd. (Shanghai, China). All mice were maintained under specific-pathogen-free conditions with a 12-h light-dark cycle and free access to rodent chow and water. Mice were randomly allocated into experimental groups (*n* =6 per group). All animal studies were approved by the Animal Ethics Committee of Shanghai General Hospital (2020AW102). Caerulein-induced AP was induced by 10 hourly intraperitoneal injections of caerulein (100 *μ*g/kg); LPS (5 mg/kg) was intraperitoneally administered immediately after the last injection of caerulein; mice were euthanized 12 h after the first caerulein injection. NaT-induced AP was induced by retrograde pancreatic ductal injection with 2.5% NaT; mice were euthanized 24 h later. Quinpirole, a DRD2 agonist, was administered intraperitoneally 0.5 h before the injection of caerulein or NaT.CA-074Me, a cathepsin B inhibitor, was administered intraperitoneally 0.5 h before the injection of caerulein or NaT.

### 2.3. Serological Test

Blood samples from mice were collected and centrifuged at 3000 rpm for 15 min at 4°C. Serum amylase, lipase, BUN, Cr, ALT, and AST levels were measured using the automated biochemical analyzer (Advia 1650, Bayer, Germany).

### 2.4. H&E Staining and Immunofluorescence

Fresh tissues were fixed in 4% neutral paraformaldehyde for 24 h at room temperature, embedded in paraffin, and then processed into 4 *μ*m slices to perform H&E staining by standard procedures. Pancreatic sections were graded from 0 to 3 for edema, inflammation, and necrosis by two blinded independent investigators.

Immunofluorescence was performed on paraffin sections. Antigen retrieval was achieved with citrate antigen retrieval solution (Sangon, #E673002) by heating the sections for 10 min. Sections were incubated with GFP antibody (Abcam, #ab6673, dilution 1 : 100) overnight at 4°C. After being washed in PBS for 3 times, sections were incubated with secondary antibody and then sealed using mounting medium with DAPI (Yeasen, #36308ES20). Confocal microscope (LEICA, SP8) was used for imaging of sections.

### 2.5. Isolation of Pancreatic Acinar Cells and Treatments

Pancreatic acinar cells (PACs) were isolated from the pancreas of C57BL6/J mice as previously described [31]. Briefly, the pancreas was injected with 2 mg/mL collagenase IV (MP Biomedicals, CA, USA) and digested at 37°C for 20 minutes. Acinar cells were carefully isolated with blunted 1-mL pipette by repeated pipetting, and then filtered through a 70 *μ*m cell strainer (Corning, Corning, NY). After being washed twice in wash buffer, PACs were resuspended in culture DME/F-12 medium containing 5% FBS and recovered for 2 h. To investigate the effect of quinpirole and CA-074Me on cell damage, PACs were pretreated with quinpirole (5 *μ*M) or CA-074Me (10 *μ*M) for 0.5 h and then stimulated with 200 nM CCK with or without LLOMe (0.5 mM) for 6 h.

### 2.6. Immunoblot Analysis and Immunoprecipitation

Total proteins were extracted from PACs and pancreatic tissues as described previously [27]. Proteins were separated by 10% or 12.5% SDS-PAGE and transferred onto nitrocellulose membranes (Millipore, CA, USA). The nitrocellulose membranes were then incubated with primary antibodies against RIP1 (1 : 1000), RIP3 (1 : 500), p-MLKL (1 : 1000), MLKL (1 : 1000), TFAM (1 : 1000), CTSB (1 : 1000), and *β*-Actin (1 : 1000) overnight at 4°C. The membranes were next incubated with HRP-labeled second antibodies for 1 h at room temperature and detected with an image analyzer using a ECL kit (Millipore, #WBKLS0100). For immunoprecipitation, isolated pancreatic acinar cells were harvested after various treatment and lysed for 30 minutes on ice, followed by a 10-minute centrifugation with the speed of 14,000 g at 4°C to collect supernatant. Protein extract was immunoprecipitated with anti-RIP1 rabbit monoclonal antibody or anti-TFAM mouse monoclonal antibody.

### 2.7. Real-Time Quantitative PCR (qRT-PCR)

Total RNA was extracted from pancreatic tissue with Trizol reagent (Invitrogen, CA, USA). RNA was reverse transcribed using PrimeScriptTM RT reagent kit (Takara Bio, Kusatsu, Japan) according to the manufacturer's protocol. Real-time quantitative PCR was performed using SYBR® Premix Ex TaqTM (Takara Bio, Kusatsu, Japan) and the applied biosystems (Life technologies, CA, USA). The relative gene expression was calculated using the comparative CT (2−*ΔΔ*CT) method with 18S as a reference gene. Primers used for qRT-PCR are listed in Supplementary Table 1.

### 2.8. Cell Injury Assays

Pancreatic acinar cell injury was measured by the percentage of ATP depletion, the activity of lactate dehydrogenase (LDH) release, and Hoechst/PI staining. Cell Titer-Glo Luminescent Cell Viability Assay Kit (Promega, Madison, WI) was used to examine according to the manufacturer's instructions. In brief, 100 *μ*L of treated-cell suspension was seeded in a 96-well culture plate and then incubated with 50 *μ*L of ATP detection reagents. The activity of LDH was assessed by LDH Cytotoxicity Assay Kit (Beyotime Biotechnology, Jiangsu, China) according to the manufacturer's instructions. In brief, 120 *μ*L of treated-cell supernatant was seeded in a 96-well culture plate and then incubated with 60 *μ*L of LDH detection reagents for 30 min. The percentage of ATP depletion and the activity of LDH were detected by a SpectraMax190 system (Molecular Devices, San Jose, CA). After being washed in PBS, PACs were stained with propidium iodide (PI; 1 mg/mL; Beyotime Biotechnology, Jiangsu, China) and Hoechst 33342 (8 mg/mL; Beyotime Biotechnology) and imaged by microscope (LEICA DMi8). Total number of cells was counted in 5 randomly selected fields. Percentage of necrosis was calculated by dividing the number of PI-positive cells by total number of cells.

### 2.9. Measurement of ROS In Vivo and In Vitro

The production of intracellular ROS in pancreas tissues and PACs was assessed with dihydroethidium (DHE) fluorescent probes. In brief, after being washed with PBS, samples were incubated using DHE for 45 min at 37°C with protection from light. Hoechst 33342 was used for nucleus staining. The fluorescence signals were detected by an inverted microscope (LEICA DMi8). Image J was used for quantification of fluorescence.

### 2.10. Adenovirus Infection

Adenoviruses were bought from OBiO Technology Corporation (Shanghai, China), including a TFAM knockdown adenoviruses using shRNA and a negative control adenovirus. Primary pancreatic acinar cells were isolated and infected with 10^7 PFU/mL adenovirus for 6 h. After being resuspended in culture medium, PACs were treated and analyzed 18 h later.

### 2.11. Adeno-Associated Virus (AAV) Injection

AAV was purchased from VIGENBIO (Shandong, China). To generate pancreas-specific TFAM knockdown, retrograde pancreatic ductal infusion of the AAV8 carrying a shRNA against TFAM was used, as previously described [32]. Briefly, after cannulating the biliary-pancreatic duct and placing a microclamp on the proximal common bile duct precisely, 100 *μ*L AAV (titer 5 X 10^12vg/mL) was infused into the biliary-pancreatic duct using an infusion syringe pump with the speed of 10 *μ*L/min (Harvard Apparatus, Holliston, MA, USA). Pentobarbital was used for general anesthesia.

### 2.12. Statistics

Statistical analyses were conducted for studies by GraphPad Prism 7.0 (GraphPad, LaJolla, CA). Data are presented as mean ± SEM. Data distribution was evaluated by the Kolmogorov–Smirnov test firstly. If data were normally distributed, parametric tests (Student's *t* test was used for two groups, one-way ANOVA for three or more groups) were carried out; if data do not follow a Gaussian distribution, nonparametric tests (the Mann–Whitney test was used for two groups, the Kruskal–Wallis test with Dunn's post-test for three or more groups) were used by GraphPad Prism 7.00 for Windows (GraphPad, LaJolla, CA). *P* < 0.05 was considered statistically significant. The results were obtained from at least three independent experiments to ensure their reliability.

## 3. Results

### 3.1. DRD2 Activation Reduced Acinar Cell Necroptosis during AP In Vivo and In Vitro

**Figure 1:**
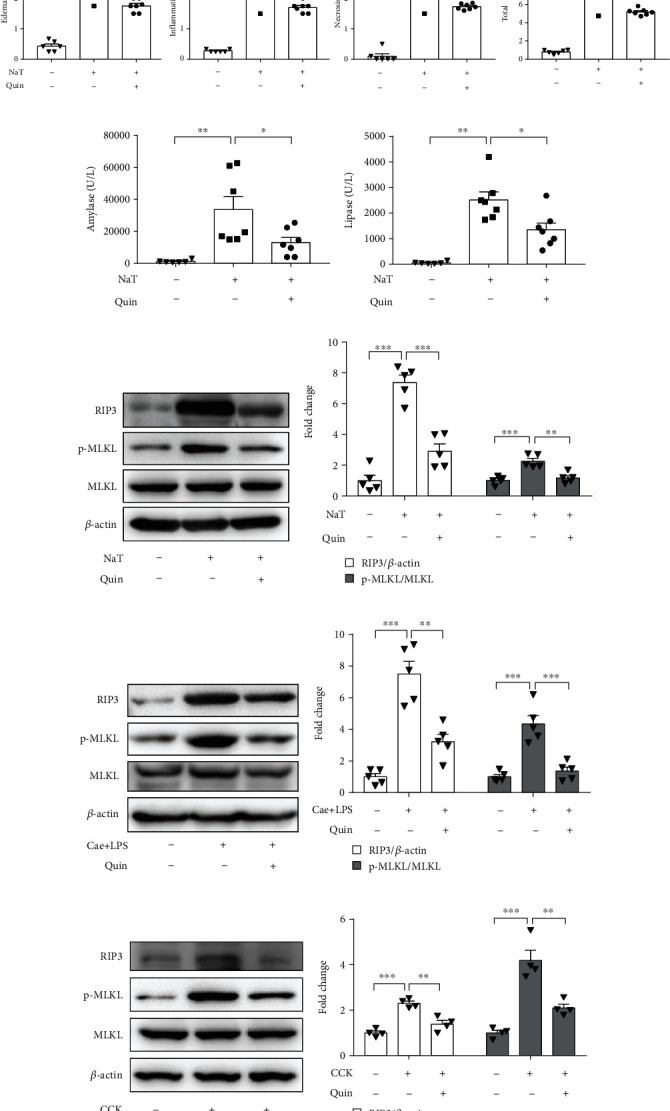
DRD2 activation with quinpirole reduced acinar cell necroptosis during AP in vivo and in vitro. In vivo AP was induced by caerulein and LPS or NaT. DRD2 agonist quinpirole was administered intraperitoneally 0.5 h before the injection of caerulein or NaT. In vitro PACs were pretreated with quinpirole (5 *μ*M) for 0.5 h and then stimulated with 200 nM CCK for 6h. (a) Representative graphs of H&E-stained pancreatic tissue (*n*=6~7 per group, scale bar 100 *μ*m). (b) Histological scores from NaT-induced pancreatitis (*n*=6~7 per group). ELISA of (c) amylase and (d) lipase in serum (*n*=6~7 per group). (e, f) Immunoblot analysis and quantification of RIP3, p-MLKL, and MLKL proteins of pancreas in mice treated with NaT (*n* =5 in each group). (g, h) Immunoblot analysis and quantification of RIP3, p-MLKL, and MLKL proteins of pancreas in mice treated with caerulein and LPS (*n* =5 in each group). (i, j) Immunoblot analysis and quantification of RIP3, p-MLKL, and MLKL proteins of pancreatic acinar cells with CCK stimulation (*n* =4 in each group). (k, l) Immunoblot analysis and quantification of CTSB proteins of pancreas in mice (*n* =3 per group). ∗*P* < 0.05, ∗∗*P* < 0.01, and ∗∗∗*P* < 0.001.

Our previous study showed that DRD2 activation mitigated pancreatic inflammation in caerulein-induced AP and L-arginine-induced AP [26, 27]. In this study, we established another experimental AP model induced by retrograde infusion of sodium taurocholate (NaT) into the mouse pancreatic duct, and as expected, DRD2 activation by quinpirole also attenuated Nat-induced AP significantly (Figures 1(a)–1(d)). In addition, quinpirole significantly reduced PAC necroptosis during experimental AP in vivo. Data showed that the protein levels of RIP3 and p-MLKL were reduced by quinpirole both in NaT-induced and caerulein-induced AP (Figures 1(e)–1(h)). Furthermore, we verified the effect of quinpirole on necroptosis of CCK-stimulated PACs in vitro. CCK stimulation upregulated RIP3 and p-MLKL levels in PACs, which were significantly reduced after quinpirole treatment (Figures 1(i) and 1(j)). Collectively, these results suggested that DRD2 activation can reduce acinar cell necroptosis during AP.

### 3.2. DRD2 Activation Alleviated Acinar Cell Necroptosis via Inhibiting CTSB In Vitro

Our previously published results demonstrated that DRD2 activation with quinpirole inhibited the expression of CTSB in experimental AP [28]. Similarly, we found that DRD2 activation with quinpirole downregulated the expression of CTSB in NaT-induced pancreatitis(Figures 1(k) and 1(l)). Accumulating evidence indicated that excessive CTSB release could shift the cell death pathway towards necroptosis [30]. Thus, we assumed that DRD2 activation reduced acinar cell necroptosis, partially through inhibition of CTSB release. We next examined the impact of CTSB on acinar cell necroptosis.

**Figure 2:**
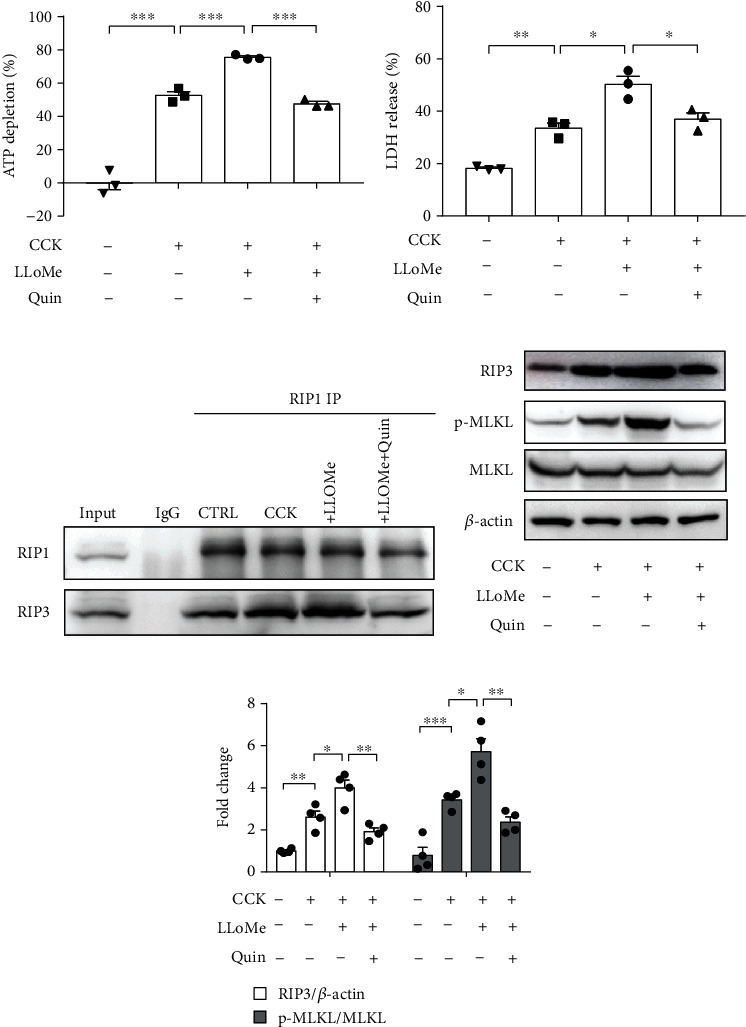
DRD2 activation with quinpirole alleviated acinar cell necroptosis via inhibiting CTSB. Isolated PACs were pretreated with quinpirole (5 *μ*M) for 0.5 h and then stimulated with 200 nM CCK with or without LLOMe(0.5 mM) for 6 h. Cell injury determined by (a) the percentage of ATP depletion and (b) LDH leakage (*n*=3 per group). (c) RIP1 was immunoprecipitated from acinar cell lysates and immunoblot analysis for RIP1 and RIP3. (d, e) Immunoblot analysis and quantification of RIP3, p-MLKL, and MLKL proteins of PACs (*n* =4 per group). ∗*P* < 0.05, ∗∗*P* < 0.01, and ∗∗∗*P* < 0.001.

Firstly, LLOMe was used to induce the release of excess CTSB from lysosomes into the cytosol in CCK-stimulated PACs, and quinpirole was used to activate DRD2. As shown in Figures 2(a) and 2(b), LLOMe exacerbated the increased ATP depletion and LDH leakage induced by CCK stimulation in PACs, whereas DRD2 activation by quinpirole partially reversed the effect of LLOMe on acinar cell necrosis. Moreover, co-immunoprecipitation assays revealed that RIP1/RIP3 necrosome was significantly enhanced after CCK stimulation. CCK-induced RIP1/RIP3 interaction was further strengthened by LLOMe, while the effect of LLOMe was reversed by quinpirole (Figure 2(c)). Furthermore, quinpirole markedly downregulated the increased protein levels of RIP3 and p-MLKL by LLOMe stimulation in PACs (Figures 2(d) and 2(e)). Taken together, these findings indicated that DRD2 activation attenuated acinar cell necroptosis partly through inhibiting CTSB.

**Figure 3:**
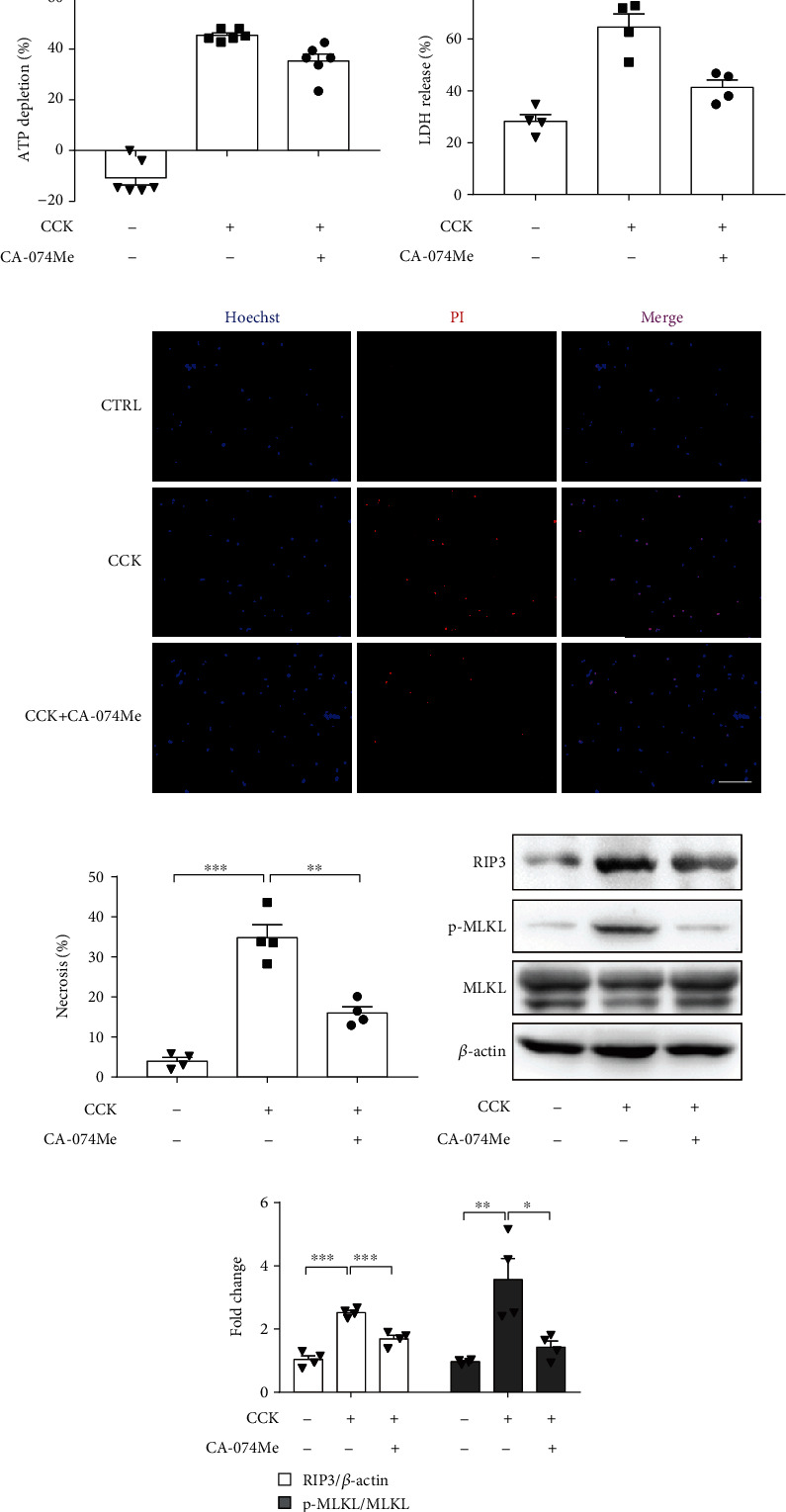
Inhibition of CTSB with CA-074Me reduced acinar cell necroptosis in vitro. Isolated PACs were pretreated with CA-074Me (10 *μ*M) for 0.5 h and then stimulated with 200 nM CCK for 6 h. Cell injury assessed by (a) the percentage of ATP depletion (*n*=6 per group), (b) LDH leakage (*n*=4 per group), and (c) Hoechst/PI staining (*n*=4 per group, scale bar 200 *μ*m). (d) Quantification of PI-positive cell (*n*=4 per group). (e, f) Immunoblot analysis and quantification of RIP3, p-MLKL, and MLKL proteins of CCK-stimulated PACs with or without CA-074Me (10 *μ*M). *N*=4 per group. ∗*P* < 0.05, ∗∗*P* < 0.01, and ∗∗∗*P* < 0.001.

Secondly, we used CTSB inhibitor CA-074Me to clarify the relationship between CTSB and necroptosis in CCK-stimulated PACs. As showed in Figures 3(a) and 3(b), we found that CA-074Me obviously prevented ATP depletion and LDH leakage induced by CCK in isolated PACs. A similar result was also presented in Hoechst/PI staining, inhibition of CTSB with CA-074Me attenuated acinar cell necrosis stained by PI (Figures 3(c) and 3(d)). Similarly, pretreatment with CA-074Me decreased the expression of RIP3 and p-MLKL in CCK-stimulated PACs (Figures 3(e) and 3(f)). Collectively, these findings indicated that inhibition of CTSB alleviated acinar cell necroptosis in vitro.

### 3.3. Inhibition of CTSB Mitigated the Severity of Experimental AP In Vivo

**Figure 4:**
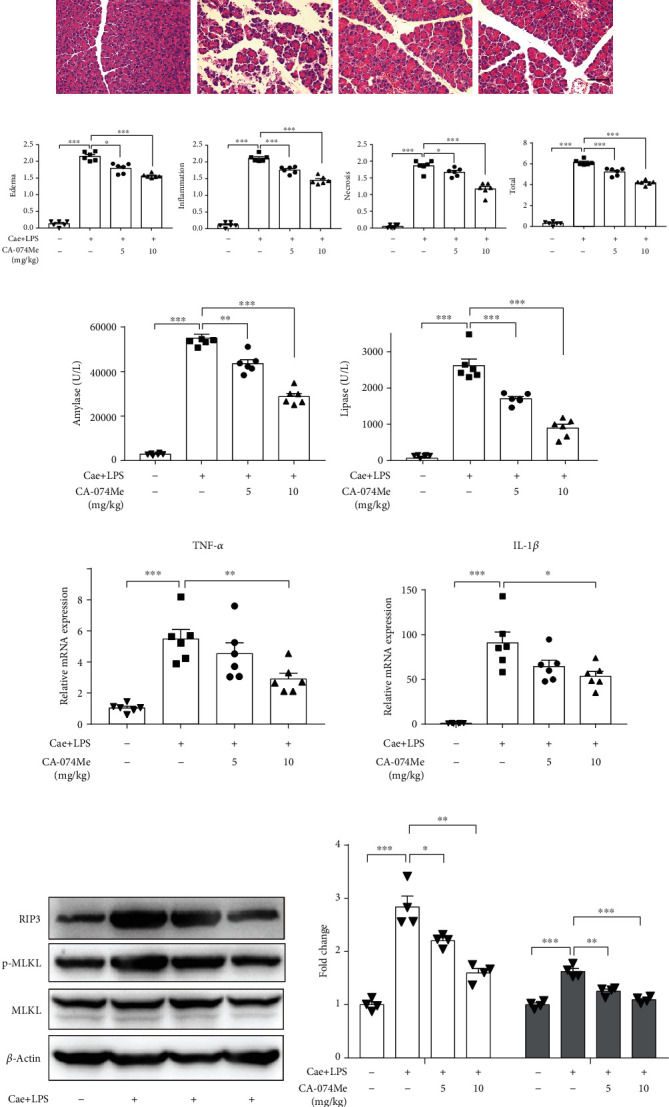
Inhibition of CTSB with CA-074Me mitigated the severity of caerulein and LPS-induced pancreatitis. AP was induced by caerulein and LPS. CA-074Me was administered intraperitoneally 0.5 h before the injection of caerulein. (a) Representative graphs of H&E-stained pancreatic tissue (*n*=6 per group, scale bar 100 *μ*m). (b) Histological scores from caerulein plus LPS-induced pancreatitis (*n*=6 per group). (c) Serum amylase and (d) lipase detected by ELISA (*n*=6 per group). (e) Expression of TNF-*α* and IL-1*β* in pancreas detected by RT-qPCR (*n*=6 per group). (f, g) Immunoblot analysis and quantification of RIP3, p-MLKL, and MLKL proteins of pancreas in mice treated with caerulein plus LPS (*n*=4 per group). ∗*P* < 0.05, ∗∗*P* < 0.01, and ∗∗∗*P* < 0.001.

**Figure 5:**
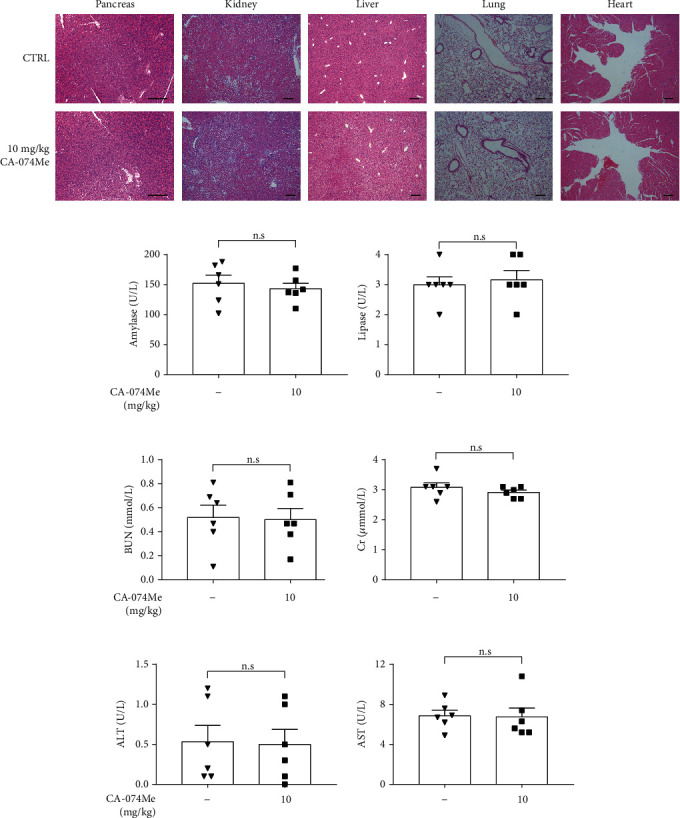
CA-074Me had no toxicity in mice. (a) Representative graphs of H&E-stained pancreas, kidney, liver, lung, and heart (*n*=6 per group, scale bar 200 *μ*m). (b) Serum amylase and lipase detected by ELISA (*n*=6 per group). (c) Serum BUN and Cr detected by ELISA (*n*=6 per group). (d) Serum ALT and AST detected by ELISA (*n*=6 per group). BUN: blood urea nitrogen; Cr: creatinine; ALT: alanine aminotransferase; AST: glutamic oxaloacetic transaminase. ∗*P* < 0.05, ∗∗*P* < 0.01, and ∗∗∗*P* < 0.001.

To further elucidate the role of CTSB in experimental pancreatitis, caerulein and LPS-induced and NaT-induced AP model were established. Firstly, in caerulein and LPS-induced AP model, mice were pretreated with different concentrations of CA-074Me (5, 10 mg/kg) before the first injection of caerulein. As shown in Figures 4(a) and 4(b), CA-074Me (at a dose concentration of 10 mg/kg) pretreatment significantly deceased pancreatic histology by assessing pancreatic edema, inflammatory infiltration, and acinar cell necrosis. Similarly, serum amylase, serum lipase, inflammatory factor of TNF-*α*, and IL-1*β* were markedly reduced in the 10 mg/kg CA-074Me-treated group (Figures 4(c)–4(e)). Moreover, RIP3 and p-MLKL levels were significantly increased during caerulein and LPS-induced pancreatitis, which were blocked by CA-074Me (Figures 4(f) and 4(g)). Of note, administration CA-074Me (10 mg/kg) alone did not show any toxicity in mice compared to control group (Figures 5(a)–5(d)). In conclusion, CA-074Me markedly mitigated AP severity and PAC necroptosis of caerulein and LPS-induced AP in vivo. Given these results, CA-074Me at a dose concentration of 10 mg/kg was adopted in the following experiments.

**Figure 6:**
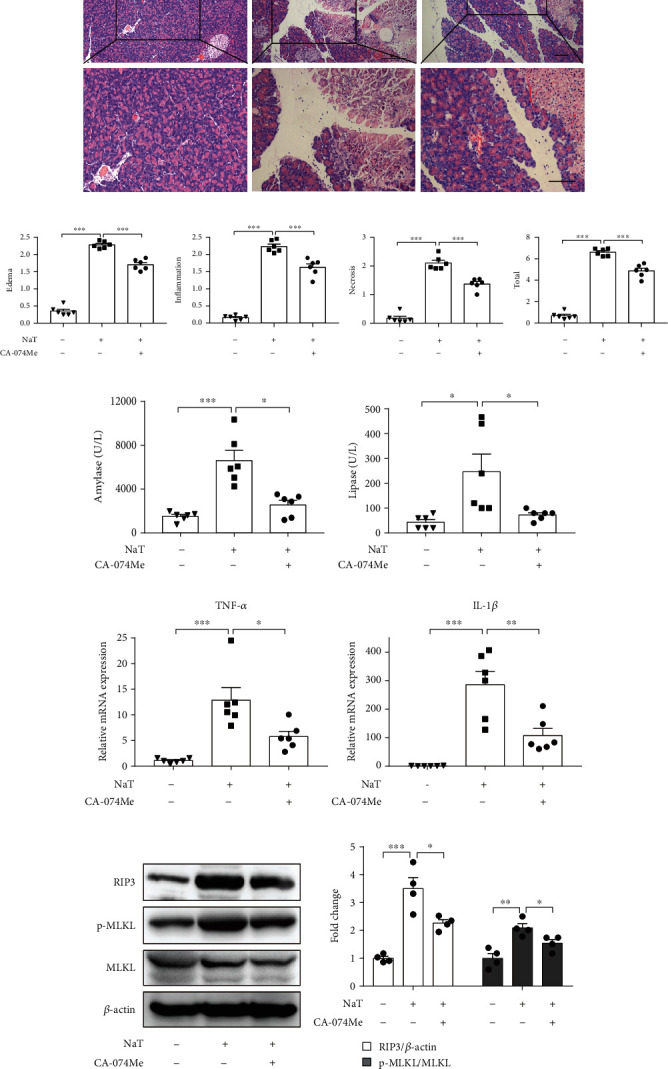
Inhibition of CTSB with CA-074Me mitigated the severity of NaT-induced pancreatitis. AP was induced by NaT. CA-074Me was administered intraperitoneally 0.5 h before the injection of NaT. (a) Representative graphs of H&E-stained pancreatic tissue (*n*=6 per group, scale bar 100 *μ*m). (b) Histological scores from NaT-induced pancreatitis (*n*=6 per group). ELISA of (c) amylase and (d) lipase in serum (*n*=6 per group). (e) Expression of TNF-*α* and IL-1*β* in pancreas detected by RT-qPCR (*n*=6 per group). (f, g) Immunoblot analysis and quantification of RIP3, p-MLKL, and MLKL proteins of pancreas in mice treated with NaT (*n*=4 per group). ∗*P* < 0.05, ∗∗*P* < 0.01, and ∗∗∗*P* < 0.001.

Secondly, NaT-induced AP model was established and treated by 10 mg/kg CTSB inhibitor CA-074Me. Consistently, we found that CA-074Me protected against NaT-induced pancreatitis, accompanied by reduced histological damages, decreased amylase and lipase levels, and downregulated the expression of TNF-*α* and IL-1*β* (Figures 6(a)–6(e)). Similar results were also observed for the protein levels of RIP3 and p-MLKL (Figures 6(f) and 6(g)). In summary, these data showed that CA-074Me markedly mitigated AP severity and PAC necroptosis both in caerulein plus LPS-induced and NaT-induced AP in vivo.

### 3.4. Knockdown of TFAM Reversed the Protective Effect of Either CA-074Me or Quinpirole during AP In Vitro

**Figure 7:**
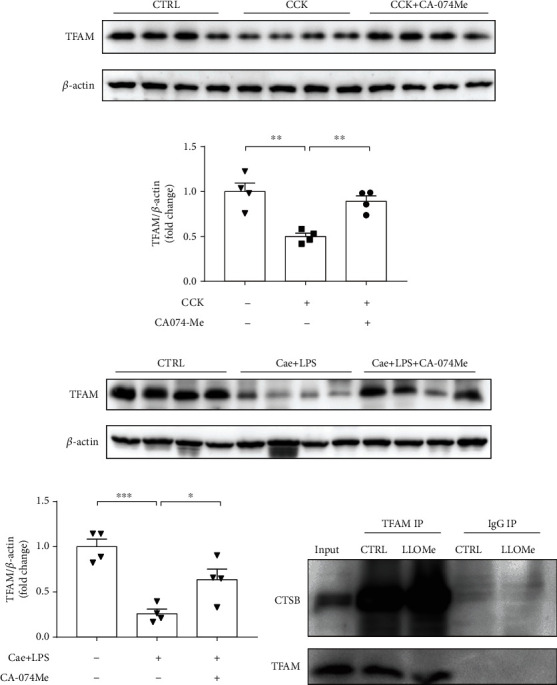
CTSB bound to TFAM and negatively regulated its expression. (a, b) Isolated PACs were pretreated with CA-074Me (10 *μ*M) for 0.5 h and then stimulated with 200 nM CCK for 6 h. Immunoblot analysis and quantification of TFAM levels of PACs with CCK stimulation (*n* = 4 per group). (c, d) AP was induced by caerulein and LPS. CA-074Me was administered intraperitoneally 0.5 h before the injection of caerulein. Immunoblot analysis and quantification of TFAM levels of pancreas in mice treated with caerulein and LPS (*n*=4 per group). (e) Isolated PACs were treated with LLOMe (0.5 mM) for 6 h. Co-ip experiment results suggested TFAM protein bound to CTSB protein. ∗*P* < 0.05, ∗∗*P* < 0.01, and ∗∗∗*P* < 0.001.

CTSB mitigated acinar cell necroptosis during AP in vitro and in vivo. However, the molecular machinery of CTSB regulated acinar cell necroptosis remains unknown. Ni et al. reported that CTSB leaked into the cytosol degraded TFAM synthesized in the cytosol, leading to oxidative stress and mitochondrial disruption in microglia during aging [33]. To explore the function of CTSB on the degradation of TFAM, we next measured the protein level of TFAM in PACs and pancreatic tissue of experimental AP. Data showed that TFAM was significantly reduced in CCK-stimulated PACs and pancreatic tissue of caerulein and LPS-induced AP, while CA-074Me reversed TFAM reduction both in vitro and in vivo (Figures 7(a)–7(d)). To further clarify the association between CTSB and TFAM, co-immunoprecipitation was used. Data showed that LLOMe stimulation enhanced CTSB and TFAM binding in PACs (Figure 7(e)).

**Figure 8:**
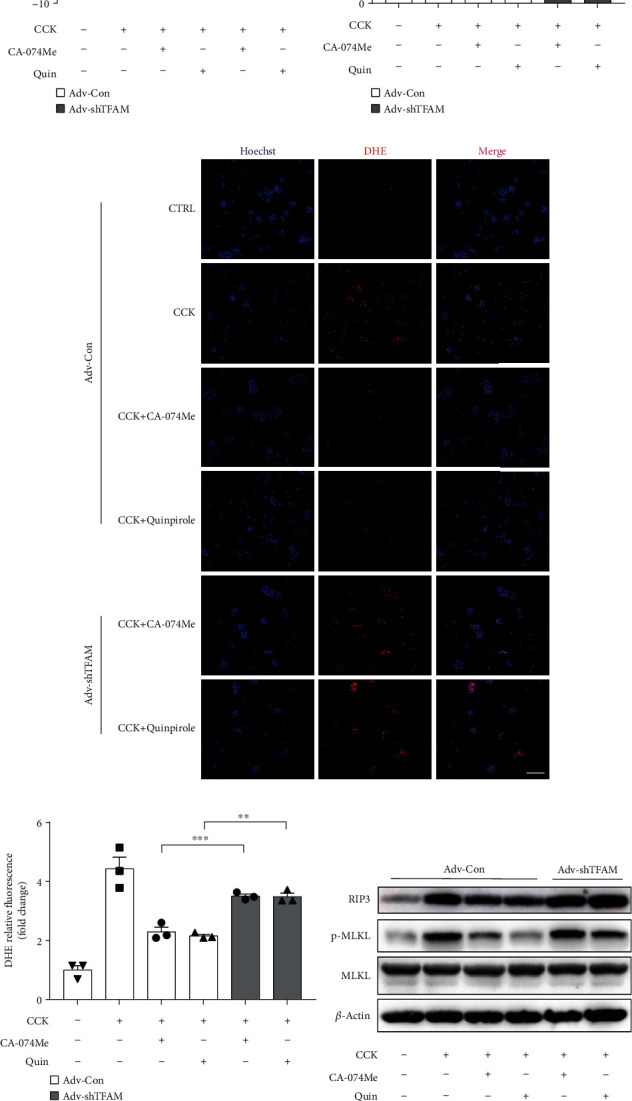
Knockdown of TFAM reversed the protective effect of either CA-074Me or quinpirole in vitro. PACs were isolated and infected with adenovirus for 6 h. After 18 h, PACs were pretreated with CA-074Me (10 *μ*M) or quinpirole (5 *μ*M) for 0.5 h and then stimulated with 200 nM CCK. (a, b) Immunoblot analysis and quantification of TFAM proteins in PACs after Adv-shTFAM infection compared to Adv-Con infection group. Cell injury determined by (c) the percentage of ATP depletion and (d) LDH leakage (*n*=4 per group). (e, f) Representative images and quantification of DHE-stained PACs showing the levels of superoxide anions (*n*=3 per group, scale bar 100 *μ*m). (g, h) Immunoblot analysis and quantification of RIP3, p-MLKL, and MLKL proteins of PACs (*n*=4 per group). ∗*P* < 0.05, ∗∗*P* < 0.01, and ∗∗∗*P* < 0.001.

To explore the effect of TFAM in PACs, we transfected TFAM-knockdown adenovirus (Adv-shTFAM) into acinar cells for 24 h and then were stimulated by CCK with or without quinpirole for 6 h. Compared with control group, the expression of TFAM was successfully knocked down to about 28% in Adv-shTFAM treated PACs (Figures 8(a) and 8(b)). Interestingly, knockdown of TFAM markedly reversed the protective effect of CA-074Me or quinpirole, as evidenced by increased ATP depletion, enhanced the release of LDH and ROS production, and upregulated the protein levels of RIP3 and p-MLKL (Figures 8(c)–8(h)). Therefore, these results suggested that CTSB modulated acinar cell necroptosis by targeting TFAM.

### 3.5. Knockdown of TFAM Reversed the Protective Effect of Either CA-074Me or Quinpirole during AP In Vivo

**Figure 9:**
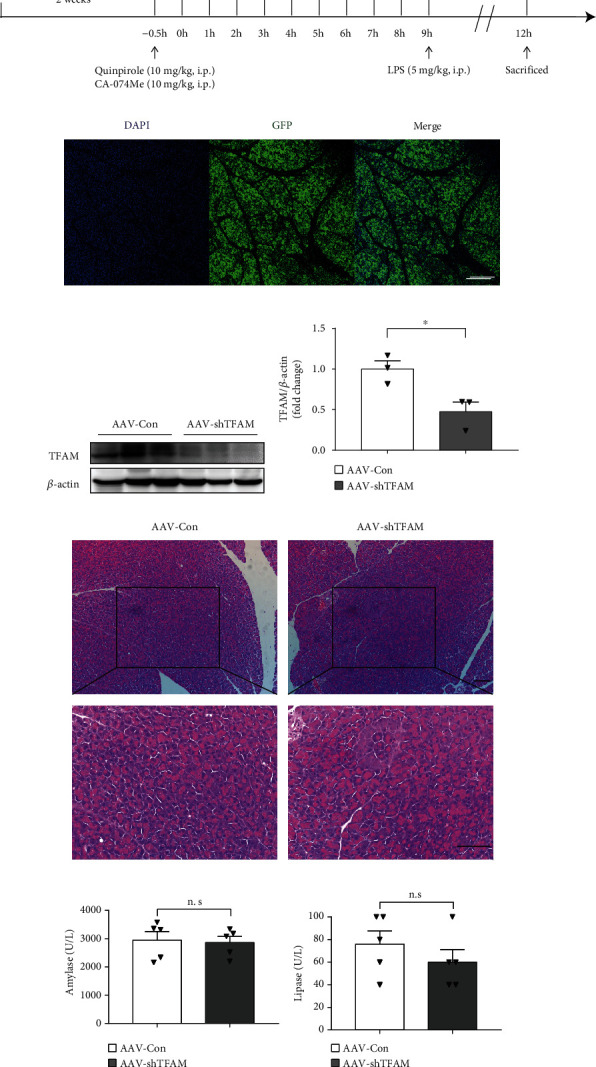
AAV injection did not cause significant local damage in pancreas. (a) The time axis of AAV injection and drug intervention in caerulein plus LPS-induced pancreatitis. (b) Representative image of GFP stained pancreas 2 weeks after AAV injection. Nuclei were stained with DAPI. (*n*=5 per group, scale bar 200 *μ*m) (c, d) Immunoblot analysis and quantification of TFAM proteins in pancreatitis after AAV-shTFAM infection compared to AAV-Con infection group (*n*=3 per group, scale bar 100 *μ*m). (e) Representative graphs of H&E-stained pancreas showing no histological changes (*n*=5 per group). (f, g) Serum amylase and lipase detected by ELISA (*n*=5 per group). ∗*P* < 0.05, ∗∗*P* < 0.01, and ∗∗∗*P* < 0.001.

**Figure 10:**
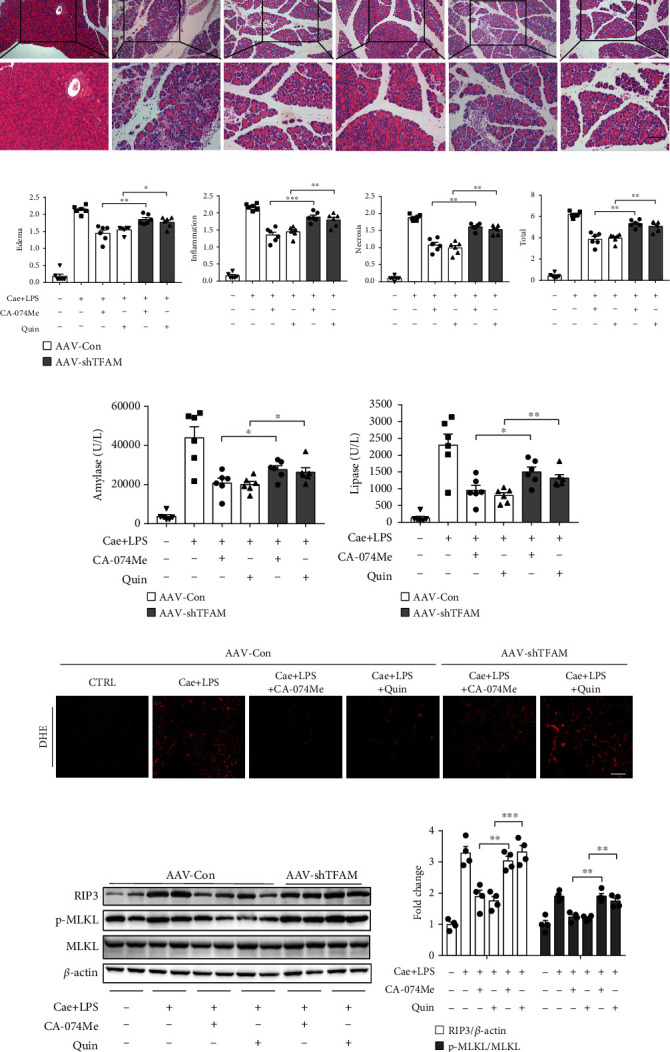
Knockdown of TFAM reversed the protective effect of either CA-074Me or quinpirole in vivo. (a) Representative graphs of H&E-stained pancreatic tissue (*n*=6 per group, scale bar 100 *μ*m). (b) Histological scores from caerulein plus LPS-induced pancreatitis (*n*=6 per group). (c) Serum amylase and (d) lipase detected by ELISA (*n*=6 per group). (e) Representative images of DHE-stained pancreas sections showing the levels of superoxide anions (*n*=4 per group, scale bar 100 *μ*m). (f, g) Immunoblot analysis and quantification of RIP3, p-MLKL, and MLKL proteins of pancreas in mice treated with caerulein plus LPS (*n*=4 per group). ∗*P* < 0.05, ∗∗*P* < 0.01, and ∗∗∗*P* < 0.001.

Adeno-associated virus (AAV) has been shown to transduce pancreatic cells in vivo efficiently without eliciting a significant inflammatory response [32]. To further confirm the role of TFAM, mice were infected with adeno-associated virus (AAV) carrying a shRNA against TFAM via pancreatic ductal infusion to knock down TFAM in the pancreas. After 2 weeks, these mice were pretreated with quinpirole or CA-074Me before the induction of pancreatitis (Figure 9(a)). The expression of TFAM was successfully knocked down in AAV-shTFAM infected pancreas compared with control group (Figures 9(b)–9(d)). HE staining and serological results showed that AAV injection did not cause significant local damage in the pancreas(Figures 9(e)–9(g)). As expected, HE staining, histological score, serum amylase, and lipase results showed that knockdown of TFAM reversed the protective effect of CA-074Me or quinpirole (Figure 10(a)–10(d)). Notably, knockdown of TFAM suppressed the effect of CA-074Me or quinpirole on ROS production (Figure 10(e)). Next, pretreatment with quinpirole or CA-074Me markedly decreased the expression levels of RIP3 and p-MLKL, while knockdown of TFAM attenuated the inhibitory effect of CA-074Me or quinpirole (Figures 10(f) and 10(g)). In summary, these results indicated that CA-074Me or quinpirole exerts a protective effect in AP via targeting TFAM.

## 4. Discussion

AP is characterized by acinar cell damage and trypsinogen activation, resulting in autodigestion of the pancreatic parenchyma, which currently has no specific treatment strategies [5]. Recent studies have shown that oxidative stress in mitochondria plays a crucial role in the development of pancreatitis [34–36]. Inhibition of mitochondrial permeability transition pore (MPTP) with cyclosporin A or genetic MPTP inhibition protects against AP biochemically and histopathologically [34]. In addition, TRO40303, a MPTP inhibitor, ameliorates alcohol-induced pancreatitis via maintaining mitochondrial function and reducing necrotic cell death [35]. Here, we found a new drug quinpirole which can restore TFAM level by inhibiting CTSB and thus attenuated acinar cell necroptosis in AP.

Our previous researches showed that DRD2 activation by quinpirole attenuated AP severity via inhibiting acinar cell NF-*κ*B activation and trypsinogen activation [26, 28]. However, the effect of DRD2 activation on acinar cell necroptosis and its underlying mechanisms remains unknown. Therefore, we established caerulein and NaT-induced AP model in vivo, and CCK-stimulated PACs in vitro, with or without DRD2 activation treatment. Consistent with previous results, this study showed that DRD2 activation alleviated AP severity in NaT-induced AP model. Furthermore, in our current complementary study, we found that DRD2 activation inhibited acinar cell necroptosis both in vitro and in vivo, which provides a new theoretical basis for elucidating AP treatment with DRD2 agonist.

CTSB is known to be associated with trypsinogen activation in acinar cells during AP [30, 37]. Intriguingly, CTSB has been implicated to be involved in regulating necroptosis depending on the high level of leakage [30]. McComb S et al. reported that CTSB can directly cleave Rip1 kinase and regulate necroptosis in macrophage [38]. We previously showed that DRD2 activation led to a marked reduction of CTSB level both in CCK-stimulated PACs and in caerulein or L-arginine-induced AP model [28]. Therefore, we assume that DRD2 activation may inhibit acinar cell necroptosis via CTSB. As predicted, we found that CTSB inhibition limited acinar cell necroptosis in this study.

Molecular mechanisms of CTSB involved in acinar cell necroptosis remain elusive. Ni et al. demonstrated that excess CTSB released into the cytosol is responsible for the degradation of mitochondrial transcription factor A (TFAM), leading to impaired mtDNA biosynthesis and increased production of mitochondria-derived ROS [33]. In this study, we found for the first time that TFAM level was decreased in caerulein-induced AP, while CTSB inhibition partly restored the level of TFAM. Of note, TFAM knockdown reversed the therapeutic effect of either quinpirole or CA-074Me. Taken together, these data suggested that TFAM is a downstream target of CTSB involved in necroptosis in acinar cell.

TFAM is a mitochondrial transcription and replication regulator encoded by a nuclear gene [39, 40]. TFAM deficiency leads to a reduction in mitochondrial DNA copy number and severe respiratory chain function defects, accompanied by a significant increase in mitochondrial ROS levels [40–42]. Mitochondrial ROS are known to promote necrotic apoptosis. Zhang et al. showed that ROS promoted necrosome formation and programmed cell necrosis by directly targeting three key cysteines RIP1, which in turn specifically enhances RIP1 autophosphorylation on S161 [16]. Consistent with these results, our data showed that pretreatment quinpirole or CA-074Me can inhibit the production of ROS and reduce necroptosis. These results shed light on CTSB/TFAM/ROS pathway for the treatment of AP served as potential targets for AP.

**Figure 11:**
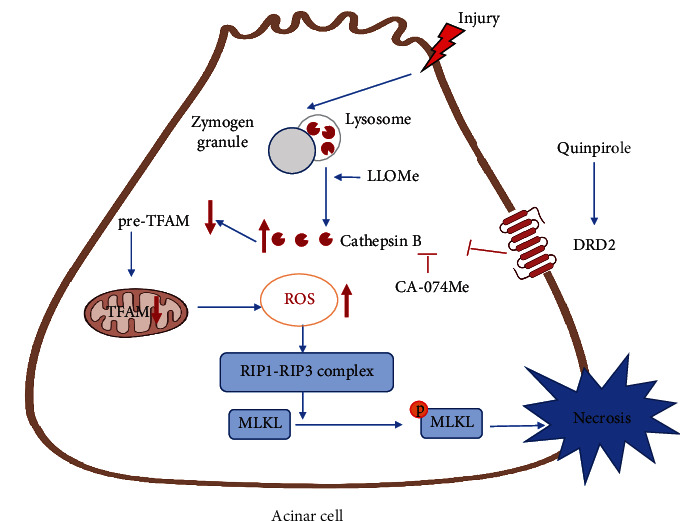
Schematic diagram summarizing the protective effects of dopamine D2 receptor on acinar cell necroptosis partly through the CTSB/TFAM/ROS pathway.

In summary, as presented in Figure 11, co-localization of zymogen granules and lysosomes occurs in PACs during AP, which promotes the release of increased CTSB from lysosomes to the cytosol. The released CTSB promotes acinar cell necroptosis through degradation of TFAM, leading to increasing intracellular ROS. DRD2 activation or CTSB inhibition ameliorated acinar cell necroptosis. Knockdown of TFAM reversed the therapeutic effect of either quinpirole or CA-074Me. Our findings provide evidence that DRD2 agonist could be a new potential therapeutic strategy for AP treatment. More broadly, these findings also shed light on the treatment for other severe inflammatory diseases.

## Data Availability

All data is available from the corresponding author by request.

## Abbreviations

AP: Acute pancreatitis
CCK: Cholecystokinin
CTSB: Cathepsin B
DRD2: Dopamine D2 receptor
LOMe: L-Leucyl-L-leucine methyl ester
MLKL: Mixed-line kinase domain-like
NaT: Sodium taurocholate
PACs: Pancreatic acinar cells
p-MLKL: Phosphorylated mixed lineage kinase domain-like
RIP1: Receptor-interacting protein 1
RIP3: Receptor-interacting protein 3
ROS: Reactive oxygen species
TFAM: Mitochondrial transcription factor A.

## Supplementary Materials

Supplementary Materials Supplementary Table 1 PCR gene primer sequences.
