# Supplementary material for: Dopamine D2 Receptor Signaling Attenuates Acinar Cell Necroptosis in Acute Pancreatitis through the Cathepsin B/TFAM/ROS Pathway
Source: Oxid Med Cell Longev. 2022 Jul 26;2022:4499219. doi: 10.1155/2022/4499219 (PMC9345736; doi:10.1155/2022/4499219)
Supplement: Supplementary Materials — Supplementary Table 1 PCR gene primer sequences. [file 4499219.f1.docx]

**Supplementary Table 1. PCR Genes Primers Sequences**

| **Gene**  **(mouse)** | **Primer sequences** |
| --- | --- |
| TNF-α Forward | 5’- TCTCTTCAAGGGACAAGGCTG -3’ |
| Reverse | 5’- ATAGCAAATCGGCTGACGGT-3’ |
| IL-1β Forward | 5’-TTGACGGACCCCAAAAGAT -3’ |
| Reverse | 5’- GAAGCTGGATGCTCTCATCTG -3’ |
| 18S Forward | 5’- CGCCGCTAGAGGTGAAATTCT -3’ |
| Reverse | 5’- CATTCTTGGCAAATGCTTTCG -3’ |
